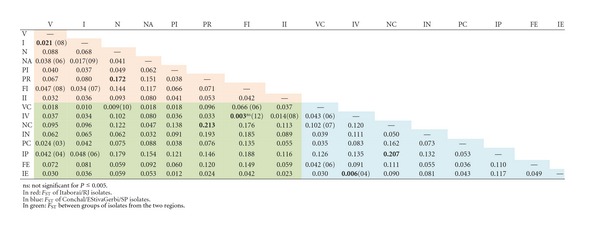# Genetic Diversity and Population Differentiation of the Causal Agent of Citrus Black Spot in Brazil

**DOI:** 10.1100/2012/368286

**Published:** 2012-05-15

**Authors:** Ester Wickert, Antonio de Goes, Andressa de Souza, Eliana Gertrudes de Macedo Lemos

**Affiliations:** ^1^Empresa de Pesquisa Agropecuária e Extensão Rural de Santa Catarina (EPAGRI), Estação Experimental de Itajaí, Rodovia Antônio Heil 8400, Itaipava, 88318-112 Itajaí, SC, Brazil; ^2^Departamento de Fitossanidade, Faculdade de Ciências Agrárias e Veterinárias de Jaboticabal, Universidade Estadual Paulista Via de Acesso Professor Dr. Paulo Donato Castellane s/n, 14884900 Jaboticabal, SP, Brazil; ^3^Departamento de Tecnologia, Faculdade de Ciências Agrárias e Veterinárias de Jaboticabal, Universidade Estadual Paulista Via de Acesso Professor Dr. Paulo Donato Castellane s/n, 14884900 Jaboticabal, SP, Brazil

## Abstract

One of the most important diseases that affect sweet orange orchards in Brazil is the Citrus Black Spot that is caused by the fungus *Guignardia citricarpa*. This disease causes irreparable losses due to the premature falling of fruit, as well as its severe effects on the epidermis of ripe fruit that renders them unacceptable at the fresh fruit markets. Despite the fact that the fungus and the disease are well studied, little is known about the genetic diversity and the structure of the fungi populations in Brazilian orchards. The objective of this work was study the genetic diversity and population differentiation of *G. citricarpa* associated with four sweet orange varieties in two geographic locations using DNA sequence of ITS1-5.8S-ITS2 region from fungi isolates. We observed that different populations are closely related and present little genetic structure according to varieties and geographic places with the highest genetic diversity distributed among isolates of the same populations. The same haplotypes were sampled in different populations from the same and different orange varieties and from similar and different origins. If new and pathogenic fungi would become resistant to fungicides, the observed genetic structure could rapidly spread this new form from one population to others.

## 1. Introduction

 Citrus black spot, caused by *Guignardia citricarpa *Kiely, is a foliage and fruit disease of citrus, affecting oranges, mandarins, lemons, and grapefruit [1]. The affected fruits become unsightly and unsuitable for the fresh fruit market, and premature fruit drop may also occur. In areas with a warm and moist climate, losses may be substantial and require intensive chemical control [2]. The fungus occurs in many areas including Asia, Australia, Southern America, and Southern Africa. It does not occur in the European Union (EU) or in the United States of America (USA), where it is considered a quarantine organism [3].

 The life cycle of this pathogen includes both asexual and sexual reproduction. Asexual pycnidiospores are disseminated from plant to plant via rain splash and despite their potential for long-distance movement, the epidemy is restricted [4]. However, the asexual stage has the potential for long-distance dissemination through the international trading of infected seeds and vegetative propagules. Ascospores produced by the sexual stage are dispersed by wind and have the potential to be blown over a considerable distance. They are not only the primary source of inoculum that could initiate an epidemy, but they also contribute to secondary infection during the growing season [5].

Although studies have been conducted on *G. citricarpa* morphology [6], disease epidemiology [7], inoculation and host response [8], and disease control [9, 10], no information has been reported about its genetic structure and if the populations of different orange varieties are genetically differentiated. The genetic structure is defined as the amount and distribution of genetic variation within and among populations, and it results from interactions among the five forces that affect the evolution of populations [11]. The genetic structure of a population is determined by the evolutionary history of that population, and knowledge of the genetic structure gives insight into the evolutionary processes that shaped a population in the past [11].

 A number of molecular techniques have been used to investigate genetic diversity and population differentiation of pathogen populations in plants. Among them, amplified fragment length polymorphism (AFLP) seems to be a more effective polymerase-chain-reaction-(PCR-) based technique than others, such as random amplified polymorphic DNA (RAPD), because it produces much more polymorphic fragments [12]. Microsatellites were used to determine genetic structure of *Botrytis cinerea* from different hosts in California [13] and RFLP markers to verify the genetic structure of *Mycosphaerella graminicola *from Texas and Switzerland [14]. The ITS1-5.8S-ITS2 cistron was used to characterise genetic diversity on *Guignardia mangiferae* [15] and verify diversity and phylogenetic relationships of *Cercospora *and *Mycosphaerella *[16].

Therefore, to obtain valuable information on the genetic structure of the *G. citricarpa *populations, we used sequence information present in the ITS1-5.8S-ITS2 region. The objectives of this study were to (i) characterise the population structure of *G. citricarpa *from different geographic regions and sweet orange varieties by determining genetic diversity and population differentiation, (ii) analyse the natural selection pressure causing genetic diversity and restriction of gene flow among populations, and (iii) analyse the possible disease management strategies associated with the genetic structure of *G. citricarpa.*

## 2. Materials and Methods

**Sampling**

The sampling was done in two different geographic areas: in the Conchal district (Coordinate 22° 19′ 48′′ S, 47° 10′ 22′′ W), located in São Paulo State, and in the Itaboraí district (Coordinate 22° 44′ 51′′ S, 42° 51′ 21′′ W), located in Rio de Janeiro State. In each place, 24 symptomatic fruits were collected, one fruit per plant, in order to obtain one isolate per plant. This was done for the four sweet Orange varieties analysed: “Natal,” “Pêra Rio,” “Valência,” and “Folha Murcha.” In the same places, in one single plant, 24 symptomatic fruits were collected in order to obtain 24 isolates from the same plant for each variety. These four varieties were chosen because they represent the most cultivated citrus trees in Brazilian orchards and are highly susceptible to CBS. On each fruit previously hygienized, three or four CBS symptoms were excised, placed on Petri plates with PDA (potato, dextrose, agar) medium and observed for appearance of *G. citricarpa* typical colonies. Colonies were then transferred to other Petri plates in order to purify the culture. Fragments of these colonies were then placed in tubes with liquid PD medium in order to produce abundant mycelium for DNA extraction.

**Culture Characterisation of *Guignardia* sp. in Oatmeal (OA) Media**

All *Guignardia* isolates from this study were characterised in oatmeal medium according to Baldassari et al. [8].

**Amplification and Sequencing of ITS1-5.8S-ITS2**

DNA from isolates was extracted according to the Kuramae-Izioka [17] protocol. Amplification of ITS1-5.8S-ITS2 was done using the primers ITS1/ITS4 [18]. PCR reactions were performed using 2 *μ*L of buffer 1X (KCl 50 mM, TRIS-HCl 200 mM pH 8,4); 0,8 *μ*L of MgCl_2_ 5 mM; 0,4 *μ*L of each dNTP 10 mM; 0,3 *μ*L Taq DNA polymerase and 5 pmol of each primer, with 60 ng of genomic DNA and sterile water q.s.p. to 20 *μ*L. DNA was amplified in Termocycler PTC-100 (Programmable Thermal Controller—MJ Research, Inc.), with 1 initial cycle at 94°C during 2 min, 39 cycles at (94°C during 1 min, 1 min at 60°C and 1 min and 30 sec at 72°C), and 1 final cycle at 72°C for 5 min. Amplified samples were separated by electrophoresis in an agarose gel (1.2%) containing ethidium bromide (0,5 *μ*g/mL) and 1 KbDNA Ladder. The samples were visualised under UV light with a GEL DOC 1000 system—Bio-Rad (data not shown). The obtained DNA fragments were purified and sequenced after PCR with the DYEnamic ET Dye Terminator Kit (GE Healthcare) according to the manufacturer's instructions. Thermocycler conditions were the same as previously described. DNA fragments were precipitated with isopropanol 75%, washed with ethanol 70%, and resuspended with 3 *μ*L of “loading buffer” (5 : 1 formamide/50 mM EDTA, pH 8.0) and denatured at 95°C during 2 min. Electrophoresis was conducted in a sequencer, *ABI Prism 3700 DNA Sequencer* (Applied Biosystems, Foster City, USA). The ITS region of each isolate was submitted to sequencing two times at both ends of each strand (Primer Forward + Primer Reverse).

**Analysis of Obtained DNA Sequences**

The electropherograms were obtained with the software *ABI Analysis Data Collection* and converted to nucleotide sequences by *DNA Sequencing Analysis Software *Version 3.3. The DNA sequences were then submitted to software Phred/Phrap/Consed [19] and Sequencher (version 4.05 (Gene Codes Corp, Ann Arbor, USA)) in order to verify sequences quality and to perform alignments and editing. All the obtained DNA sequences were submitted to GeneBank-NCBI for comparing with the deposited sequences by the BLAST tool [20]. The aligned sequences were then used for the subsequent analysis, by the detection of SNPs.

**Intra- and Intergroup Genetic Distances**

Genetic distances were calculated between groups of pathogenic isolates from the same plant, from different plants, and from different geographic origins. These estimates were calculated in order to evaluate the genetic diversity among the intra- and intergroup, according to Nei's equations [21]. The intragroup genetic distance was estimated by the arithmetic mean of the distance between each of the isolates, compared in pairs [22]. The intergroup distances were calculated for groups of different plants and different geographic origins as the arithmetic average of all the distances between the two analysed groups [22]. These values were calculated with Kimura-2-Parameter [23] with the software, MEGA (version 3.1) [24].

**Nucleotide and Haplotype Diversity**

Average pairwise differences were estimated from comparisons within a library of the number of sequence differences between a given clone and all other clones [25] (Table 5). To estimate genetic diversity within the two libraries, some indices were calculated using the distance method with a Kimura-2-parameter substitution nucleotide model. Average pairwise differences and nucleotide diversity were calculated for each library. Also molecular indices like number of gene copies and haplotypes, total number of loci, usable loci, polymorphic sites, and gene diversity were estimated for each data set. Nucleotide diversity was estimated from the number of variable positions for aligned sequences in a given library.

**Genetic Differentiation (*F*_ST_) and Gene Flow (Nm)**

*F*_ST_ values were used to evaluate the genetic diversity within the groups of isolates in relation to the total genetic diversity according the equation *F*_ST_ = (*θ*_*T*_ − *θ*_*W*_)/*θ*_*T*_, where *θ*_*T*_ is the genetic diversity of all isolates and *θ*_*W*_ is the diversity within the group of isolates [26]. Analysis of molecular variance (AMOVA) was performed using Arlequin version 3.0 [27]. Population structures were defined on the basis of phylogenetic clusters that we obtained. A hierarchical analysis of variance was carried out to partition total variance into variance components attributable to interindividual and/or interpopulation differences. Variance components were then used to compute fixation indices, and their significance was tested at 1,000 permutations, as described by Excoffier et al. [25]. Gene flow was calculated by the number of migrants per generation (Nm) according to equation 4 of Hudson et al. [28] by the software DNAsp, version 4.50.3 [29].

**Genetic Relationships**

The aligned sequences were used to verify the genetic relationships among the isolates from same and different sweet Orange varieties from the two places. Dendrograms were built using the Distance Method and grouped by the algorithm, Neighbour Joining [30] and the nucleotide substitution model Kimura-2-parameter [23] with the software MEGA (Versão 3.1) [24]. Method reliability was calculated by bootstrap values [31] with 1,000 repetitions by the same software. Dendrograms were built to observe the similarity within the groups of isolates and with the Guignardia DNA sequences from different species obtained from GenBank. The Guignardia ITS1-5.8S-ITS2 DNA sequences included in each analysis file were G. citricarpa clone 75 (ID:AF346782.1); G. citricarpa (ID:AF346772.1); G. mangiferae voucher ICMP 8336 (ID:AY816311.1); G. mangiferae (ID:AM403717.1); G. laricina (ID:AB041245.1); G. philoprina (ID:AB095507.1); G. philoprina specimen-voucher CBS 447.68 (ID:AF312014); G. aesculi (ID:AB095504.1); G. vaccinii (ID:AB041244.1); G. bidwellii (ID:AB095511.1); G. bidwellii (ID:AB095505); G. bidwellii (ID:AB095509); G. gaultheriae (ID:AB095506.1); G. gaultheriae (ID:AB095506); Phyllosticta pyrolae (ID:AF312010); Phyllosticta pyrolae (ID:AB041242); Phyllosticta spinarum (ID:AF312009).

**Pathogenicity Tests**

Pathogenicity tests were conducted according Baldassari et al. [8] using 22 isolates from Estiva Gerbi/Conchal/SP (3 isolates from VC group, 2 isolates from IV group, 4 isolates from NC group, 2 from IN group, 3 from PC group, 3 from IP group, 3 from FE group, and 2 from IE group). The isolates were inoculated on sweet Orange “Pêra” in January/February of 2007. Fruits were harvested in September 2007 and evaluated for the presence/absence of classic symptoms of CBS.

## 3. Results

**Sampling**

*Guignardia* typical colonies were obtained from all the varieties and geographic origins, in a total of 384 isolates. All samples, in the same and different plants in the 4 orange varieties and two different places, were composed of 24 isolates (Table 1).

**Culture Characterisation of *Guignardia* sp. in Oatmeal (OA) Media**

**Figure 1:**
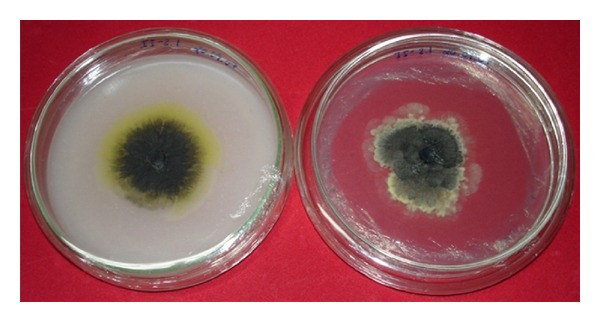
Aspects of *G. citricarpa* colony morphology in oatmeal medium (left), showing the yellow halo, characteristic for pathogenic isolates, and its aspect on PDA medium, without halo.

All 384 *Guignardia* isolates submitted to characterisation in oatmeal media showed a yellow halo around the colonies (Figure 1) that is indicative of the *G. citricarpa* species, pathogenic to citrus plants [2, 8]. This method thereby ensures that all isolates of this study effectively belong to the *G. citricarpa* species.

**Amplification and Sequencing of ITS1-5.8S-ITS2**

DNA from the isolates was used to amplify the ITS1-5.8S-ITS region. All isolates showed a characteristic band of approximately 800 bp in an agarose gel. When submitted to sequencing, all isolates showed a fragment with an approximately 780 bp length.

**Analysis of DNA Sequences**

The obtained sequences were submitted to a quality analysis in order to use only those that displayed high quality. All sequences showed the desired quality by software Phred/Phrap/Consed. This was done in order to prevent mistakes during later analysis. None of the sequences showed apparent heterozygotes in this region. All sequences were submitted to GenBank (http://www.ncbi.nlm.nih.gov/genbank) and its ID are showed in supplementary material available online at doi:10.1100/2012/368286.

**Intra- and Intergroup Genetic Distances**

All isolates showed small genetic distances, indicating high genetic similarity. When all isolates from the same geographic region were analysed as one single group (intragroup), Itaboraí isolates presented a genetic distance slightly lower (0.01527) than the group of isolates from Estiva Gerbi/Conchal (0.01714). The genetic distance between (intergroup) all isolates from these two regions was 0.01637.

Isolates from Estiva Gerbi/Conchal, obtained from a single plant of “Folha Murcha” presented the highest intragroup genetic distance (0.02500), whereas the lowest was presented by the “Natal” variety (0.00972) from Itaboraí (Table 1).

When Itaboraí isolates where analysed, the isolates from the same plant of the “Folha Murcha” variety presented the highest intragroup genetic distance (0.02027). The lowest intragroup distances were presented by isolates from different plants of the “Natal”variety (0.00972) (Table 1). In Itaboraí, isolates from “Valência”, “Natal” and “Folha Murcha” presented the higher genetic distances among the groups of isolates from the same plant as compared to isolates collected in different plants of these varieties. Only the “Pera” variety showed greater genetic diversity among isolates from different plants when compared to isolates from the same plant.

Among the isolates from Estiva Gerbi/Conchal, the isolates from a single plant of the “Folha Murcha” variety presented the higher intragroup genetic distance (0.02500), and the lower distance was represented by isolates from a single plant of the “Natal” variety (0.01025) (Table 1). In EstivaGerbi/Conchal, isolates from “Valência” and “Folha Murcha” also presented the higher genetic distances among the groups of isolates from the same plant than isolates collected in different plants of these varieties. The “Pera” and “Natal” varieties showed the higher genetic diversity by isolates sampled from different plants than from isolates of the same plant.

The highest intergroup genetic distances were represented by isolates from the same geographic origin, EstivaGerbi/Conchal, by single-plant isolates of “Folha Murcha” and “Valência” (0.02361) (Table 2). Among the sixteen studied populations these two populations can be considered as having the highest genetic divergence.

The lowest divergence was represented by groups of isolates from different geographic origins, by isolates from “Natal” of different plants from Itaboraí and “Valência” and different plants from Estiva Gerbi/Conchal (0.01049). These two populations can be considered as having lowest genetic diversity.

**Nucleotide and Haplotype Diversity**

These diversity indices showed that the highest genetic diversity was found for the groups of isolates from São Paulo state, IE and IV, from the same plant of “Folha Murcha” and “Valência” varieties, respectively (Table 3). These two groups of isolates showed the highest number of polymorphic sites, mean number of pairwise distances, and nucleotide diversity, with each sequence representing one haplotype for the IV group. For the IE group, 21 haplotypes were found among the 24 isolates. The lowest genetic diversity was also found in São Paulo state for the groups of isolates from the same and from different plants of the “Pêra” variety, IP and PC. These two groups presented the lowest number of polymorphic sites, mean number of pairwise distances, and nucleotide diversity, with one haplotype representing more than one sequence. The group of PR isolates from Itaboraí/RJ obtained from a single plant of the “Pêra” variety presented the lowest number of haplotypes within the group, but intermediate values for the other indices, probably because of the presence of different nucleotides in the same position among different sequences.

**Genetic Differentiation (*F*_ST_) and Gene Flow (Nm)**

According to *F* values, little genetic differentiation among *G. citricarpa *populations was observed at various hierarchical levels (among regions, among populations within regions, and within populations) (Table 4). The analysis of the ITS1-5.8S-ITS2 DNA sequence indicated that genetic differentiation of *G. citricarpa *within each sample was significant (*F*_ST_ = 0.09894, *P* ≤ 0.0001), representing 90.86 percent of the observed genetic diversity. The fixation index among populations within regions was also significant (*F*_SC_ = 0.09143, *P* ≤ 0.0001), representing 9.98 percent of the observed genetic diversity. The fixation index among the two regions is almost insignificant (*F*_CT_ = −0.00834, *P* ≤ 0.0001), indicating that there is gene flow between regions.

When differentiation indices were calculated for groups of isolates according to the orange variety (Table 5), we observed that in Itaboraí/RJ, the highest differentiation was observed for “Pêra” and “Natal” (*F*_SC_ = 0.102, *P* ≤ 0.005). The lowest was represented for “Valência” and “FolhaMurcha” (*F*_SC_ = 0.031, *P* ≤ 0.005). In Estiva Gerbi/Conchal/SP, the highest differentiation was also observed for “Pêra” and “Natal” (*F*_SC_ = 0.127, *P* ≤ 0.005), and the lowest was observed for “Valência” and “FolhaMurcha” (*F*_SC_ = 0.017, *P* ≤ 0.005). Between the two regions, “Pêra” and “Natal” showed the highest genetic differentiation (*F*_SC_ = 0.141, *P* ≤ 0.005), and “Valência” and “FolhaMurcha” represented the lowest (*F*_SC_ = 0.005, *P* ≤ 0.005, not significant). When we analysed the same orange variety from both places, the highest population differentiation was displayed by the “Pêra” variety (*F*_SC_ = 0.087, *P* ≤ 0.005) and the lowest by “Valência” (*F*_SC_ = 0.031, *P* ≤ 0.005).

Genetic differentiation was also estimated for the groups of isolates according to variety, sampling, and geographic origin (Table 6). In Itaboraí/RJ, the highest differentiation was observed for the “Pêra” population of the same plant and the “Natal” population of different plants (PR and N) *F*_ST_ = 0.172, *P* ≤ 0.005) and the lowest for “Valência” populations from the same and different plants (V and I) (*F*_ST_ = 0.021, *P* ≤ 0.005). In Estiva Gerbi/Conchal/SP, the highest differentiation was also observed for the “Pêra” population of the same plant and the “Natal” population of different plants (IP and NC) (*F*_ST_ = 0.207, *P* ≤ 0.005) and the lowest for “Valência” and “Folha Murcha” populations from the same plant (IV and IE) (*F*_ST_ = 0.006, *P* ≤ 0.005). When populations of the two places were compared, the highest differentiation was by the “Pêra” population of the same plant and the “Natal” population of different plants (PR and NC) (*F*_ST_ = 0.213, *P* ≤ 0.005); the lowest differentiation was represented by the “Valência” population from the same plant and “FolhaMurcha” populations from different plants (IV and FI) (*F*_ST_ = 0.003, *P* ≤ 0.005, not significant).

The analysed populations also shared haplotypes (Table 6), and they were found between populations of the same variety from the same geographic origin, between different varieties of the same geographic origin and between different varieties from two different geographic origins. The highest number of shared haplotypes was displayed by the “Valência” population from the same plant and the “Folha Murcha” populations from different plants (IV and FI) (12 shared haplotypes).

Gene flow was also detected among the studied populations (Table 7). All sampled populations represented gene flow at different levels. The highest level of gene flow between populations from Itaboraí/RJ was detected between populations of the same and different plants of “Valência” (V and I, 88.31 migrants per generation), and the lowest was detected between the “Natal” population of different plants and the “Pêra” population of the same plant (N and PR, 1.66 migrants per generation).

In Estiva Gerbi/Conchal/SP, the highest level of gene flow between populations was detected between the sample of different plants of “Valência” and different plants of “Folha Murcha” (VC and FE, 11.12 migrants per generation) and the lowest between the “Valência” population of the same plant and the “Pêra” population of the same plant (IV and IP, 1.34 migrants per generation). The highest levels of gene flow were seen when populations from the two geographic origins were compared (FI and IV, 133.01 migrants per generation; N and VC, 112.15 migrants per generation).

**Genetic Relationships**

**Figure 2:**
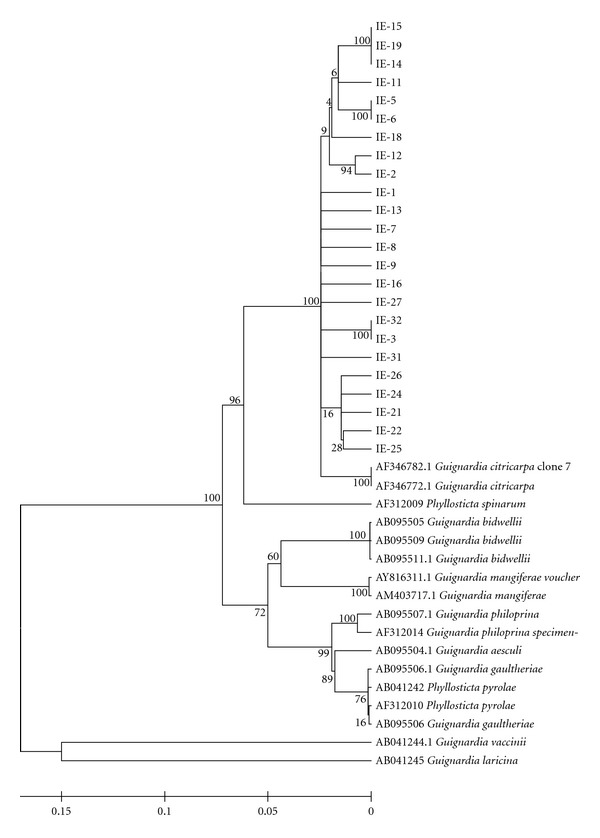
Genetic relationships inferred by ITS1-5.8S-ITS2 DNA sequence from isolates obtained in fruits from a same plant with CBS symptoms of “Valência” variety in Conchal/SP. It can be verified that all isolates show high similarity to each other and with the *G. citricarpa* sequence obtained in GenBank. High divergence was found with the obtained isolates and GenBank sequences of *G. laricina* and *G. vaccinii*.

**Figure 3:**
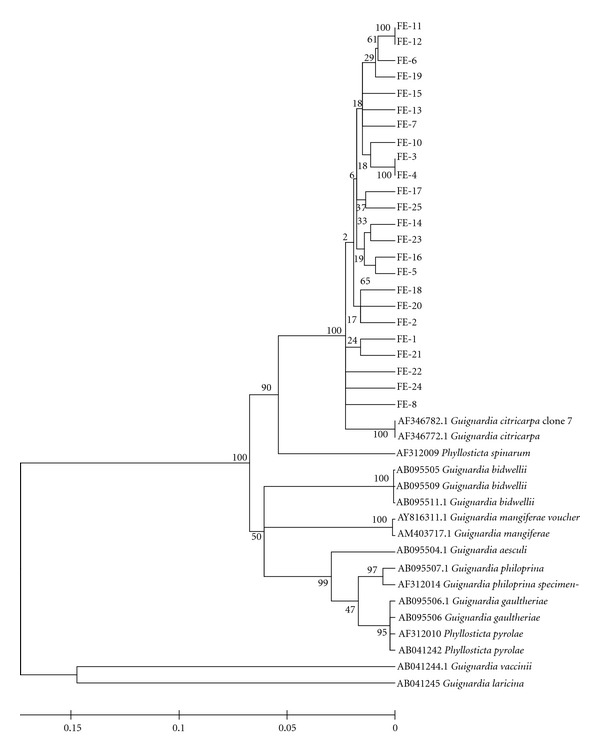
Genetic relationships inferred by ITS1-5.8S-ITS2 DNA sequence from isolates obtained in fruits from different plants with CBS symptoms of Conchal/SP. It can be verified that all isolates show high similarity to each other and with the *G. citricarpa* sequence obtained in GenBank. High divergence was found with the obtained isolates and GenBank sequences. The nearest sequence to *G. citricarpa* belongs to *P. spinarum*.

The 16 obtained populations were analysed to verify the similarity of the isolates of one population to another and to the sequences obtained from GeneBank. All isolates grouped to *G. citricarpa* sequences obtained from GeneBank displayed great similarity between each other, as is exemplified by populations obtained from the same (IE, Figure 2) and different plants (FE, Figure 3) of the “Folha Murcha” variety of Estiva Gerbi/Conchal/SP. All studied populations presented similar grouping and belonged to the *G. citricarpa* species. The other *Guignardia* species used to compare with the obtained isolates was not closely related. The most related GeneBank sequence to *G. citricarpa* isolates was *Phyllosticta spinarum*, whose teleomorphic form was unknown until now.

**Pathogenicity Tests**

**Figure 4:**
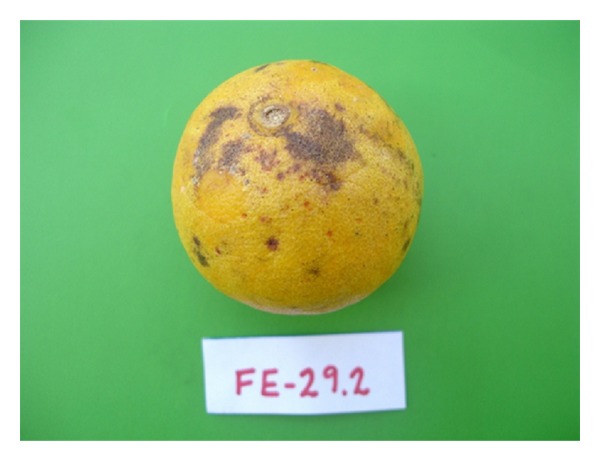
Aspect of fruit inoculated with *G*. *citricarpa* isolate showing the characteristic symptoms of CBS.

Pathogenicity tests were conducted in order to verify if the obtained isolates could cause disease in inoculated fruits. All 22 isolates caused symptoms in fruits, mainly with freckled and hard spots (Figure 4).

## 4. Discussion

We performed a study of genetic variation and population differentiation of an important pathogen for Brazilian citriculture, *G. citricarpa*, from a large geographical area that covers the oldest and highly productive areas of citrus in Brazil. The DNA sequences of *G. citricarpa* ITS regions were found to contain adequate levels of genetic variation to assess its genetic diversity and population differentiation. Despite the fact that the majority of published studies about population structure did not use only these sequences to estimate population differentiation indices, we believe that, in this case, the obtained results agree with previous studies about etiology and epidemiology of these fungi. These fungi species cause severe losses to almost all cultivated citrus varieties, and, as far as we know, this is the first report on *G. citricarpa* population diversity and differentiation.

In this study, the sequence information was used to identify SNP markers to detect genetic variation and revealed a low degree of genetic variability within and among the sixteen studied populations. The diversity indices for the *G. citricarpa* populations in the two geographic areas showed similar results, with few differences among the four studied orange varieties, which are the most cultivated in Brazilian citriculture. As AMOVA analysis showed, the main diversity (90.86%) was found within the populations. A minor diversity was found within regions (9.98%) and can be credited possibly to influences of the host over the populations. Little genetic diversity was found between the two sampled sites, showing that the same or similar haplotypes were found in all populations, despite the fact that the two geographical areas are distant from one another (around 650 km) and have different climatic and soil conditions.

*G. citricarpa* also displayed no traits inherent to a specific pathogen to any of the studied orange varieties, with a high number of haplotypes being shared by different populations of the same and different orange varieties and by populations of the two different geographic areas. As the two areas where the populations were collected are not close to one another geographically and citriculture is widespread between the two areas, this can facilitate gene flow among populations. The diversity indices also showed that sampling done in the same plant presented similar genetic diversity as the sampling conducted in different plants, probably because one plant can host different *G. citricarpa* haplotypes.

 The coexistence of different pathogen haplotypes within the same host plant, as detected by this work, has diverse biological implications beyond the increased opportunities for sexual reproduction. Colonization of the host by different genotypes of the same pathogen leads to an increase of within-host competition and a selection of higher pathogen virulence [32].

 We believe that the gene flow is not restricted between populations in the same geographic area or in populations from the two geographic areas because citriculture is widespread in Brazil. *G. citricarpa* populations separated by thousands of kilometres were genetically similar, as indicated by low population differentiation and high genetic identity. The low levels of population differentiation were reflected in corresponding high values of Nm (gene flow). Despite the fact that low *F*_ST_ values may arise from gene flow as well as recent population expansion even in the absence of gene flow [33], we believe that this case is caused primarily by the existence of gene flow.

 Low levels of population differentiation and corresponding high levels of genetic similarity suggest that gene flow has had a significant impact on the genetic structure of these populations [14]. In light of the low *F*_ST_ values among populations from different geographic areas and also among populations of the same area, we believe the mechanism of dispersal is at work in *G. citricarpa.* Propagation structures in *G. citricarpa *are either sexually formed ascospores or asexual pycnidia. The fungal spores generated by mitosis (“conidia”) formed inside specialised organs (“pycnidia”) are frequent in *G. citricarpa *and are found on fruit lesions during the ripening stage, but they are unlikely to function as dispersal units over long distances [34]. Ascospores, whether formed by a homo- or heterothallic process, are tiny and may disperse over relatively short and large distances [35], whereas pycnidia are large and heavy and likely to disperse primarily over short distances [34]. So, it is supposed that ascospores are responsible by the epidemic, whereas conidia are responsible for the disease development on the plant [36, 37].

 If pycnidia had been abundant at the particular spatial scale in our study, we would have expected to see possibly higher within-habitat *F*_ST_ values. In the absence of such observations, evidence seems to favour ascospores as the common dispersal unit at the present spatial scale. However, an alternative explanation may be that wind or human activities acted as vectors for dispersing pycnidia over larger distances than expected.

As is common with most organisms, plant-pathogenic fungi rely on the processes of mutation and recombination as the ultimate source of genetically based variation. Within a species, gene flow between populations supplements these processes as propagules spread from one epidemiological area to another and from one deme to the next [38].

The major focus of population genetics is to understand the evolutionary processes shaping and maintaining the distribution of genetic variation distributing within and among populations [39]. The definition of the genetic structure of populations is a logical first step in studies of fungal population genetics because the genetic structure of a population reflects its evolutionary history and its potential to evolve [11]. For evolution to occur by natural selection there must be variation in fitness among individuals. Fisher's fundamental theorem of natural selection states that the mean fitness of a population is always increasing and that the rate of increase is proportional to the amount of additive genetic variation in fitness in a population [11]. In more general terms, Fisher's theorem says that the evolutionary potential of a population is proportional to the amount of genetic diversity in it [40]. According to this, it is hypothesised that knowledge of genetic structure also offers insight into the future evolutionary potential of pathogenic populations [11].

In Brazilian citriculture, the current and regular use of fungicides can cause a selective pressure over the *G. citricarpa* populations, changing its structure and increasing the probability of selecting resistant strains to the fungicide's active molecules. Selection or emergence of resistant strains can be done in Brazil by an increase of selection pressure and because the teleomorph and anamorph phases of *G. citricarpa* are currently found in affected Brazilian orchards. Given the little genetic differentiation among the studied populations, this suggests frequent recombination events [41], we can infer that there are not strong physical barriers to gene flow in Brazil because there are citrus orchards distributed over the entire studied area and the constant winds can contribute to the spread of ascospores over great areas. In Brazilian environmental conditions and panmictic populations, as detected in this work, the emergence and spread of new and more aggressive or fungicide-resistant pathotypes could be efficient and very fast [41].

 Dusabenyagasani et al. [42] made similar estimates of both population diversity and interpopulation similarity for Canadian populations of *Gibberella zeae *(*G*_ST_ < 0.05), also suggesting that these populations were panmictic. Low levels of *G*_ST_ also have been reported between populations of other plant-pathogenic fungi, including *Rhizoctonia *[14], *Rhynchosporium *[43], and *Botrytis cinerea* [13], and in these cases, the authors also concluded that these fungi exist as larger, well-mixed populations.

 Despite being collected in different years and from sites over 650 km apart, the low values for *F*_ST_ (<0.01 among the two sites) and an estimate of very high genetic identity (nearly equal to 1.0) both suggest that these sixteen *G. citricarpa* populations are part of a much larger, probably panmictic, pathogen population covering much of the Brazilian Southeastern.

 The likelihood that high levels of gene flow occur on a regional scale indicates a substantial risk for the regional spread of mutant alleles that enable the breakdown of resistance genes or fungicide-resistance. Since the mutant allele travels regionally in ascospores (gene flow) instead of conidia (genotype flow), the mutant allele will move between fields in a recombined genetic background that has not been preselected for a highly fit combination of coadapted alleles. As a result, the development of new virulent pathotypes or fungicide resistant strains may in many cases be gradual rather than abrupt [11]. In this way, the gene flow on a regional level could be reduced by strategies that minimise the production of ascospores [14], such as improved management of the fallen citrus orange leaves.

 The management of fallen citrus orange leaves has been highly recommended to Brazilian citrus producers. This has been done by recommending the cultivation of species like *Crotalaria* sp. and *Cajanus cajan* between the citrus lines. The use of a rotary cutter permits the covering of the fallen citrus leaves and makes the spread of ascospores more difficult. The use of these cultures between citrus lines also has the advantage of increasing soil nitrogen levels and reducing the fall of the citrus leaves.

## Supplementary Material

This Table contains the *Guignardia mangiferae* isolates used for this study. ITS1-5.8S-ITS2 DNA region of each isolate was amplified in order to perform the population genetic structure described on this research. The DNA sequence was deposited in GenBank and received an Acession Number presented on Table.

## Figures and Tables

**Table 1:** Number of isolates by sampling and intragroups genetic distances showed by groups of isolates from symptomatic tissues from the four different orange varieties in two geographic places.

Geographic origin	Isolates	Number of isolates	Intragroups distance
Itaboraí/RJ	V—Valência/different plants	24	0.01395
	I—Valência/same plant	24	0.01609
	N—Natal/different plants	24	**0.00972**
	NA—Natal/same plant	24	0.01474
	PI—Pêra/different plants	24	0.01498
	PR—Pêra/same plant	24	0.01276
	FI—Folha Murcha/different plants	24	0.01274
	II—Folha Murcha/same plant	24	**0.02027**

Estiva Gerbi/Conchal/SP	VC—Valência/different plants	24	0.01105
	IV—Valência/same plant	24	0.02191
	NC—Natal/different plants	24	0.01717
	IN—Natal/same plant	24	**0.01025**
	PC—Pêra/different plants	24	0.01167
	IP—Pêra/same plant	24	0.01041
	FE—Folha Murcha/different plants	24	0.01919
	IE—Folha Murcha/same plant	24	**0.02500**

**Table 2:** Intergroups genetic distances showed by the groups of isolates from CBS symptoms in four orange varieties and two geographic origins.

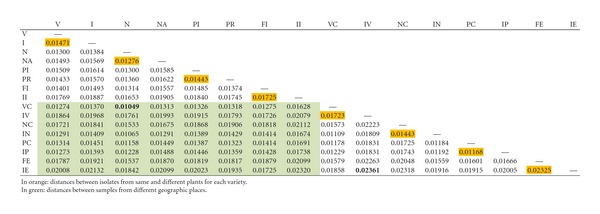

**Table 3:** Diversity indexes calculated for 16 populations of *G. citricarpa* from four orange varieties and two different geographic origins.

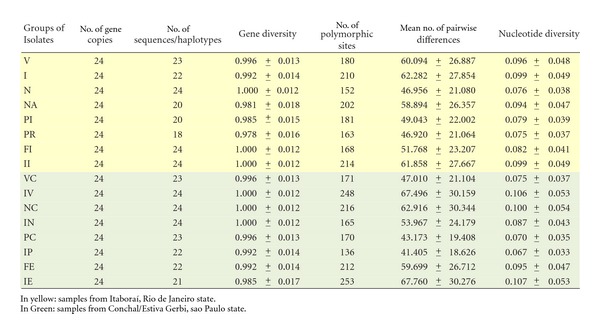

**Table 4:** AMOVA analysis comparing results of genetic variation from *G. citricarpa *sampled in four sweet orange varieties in same and in different plants in two geographic places.

Source of variation	d. f.	Sum of squares	Variance components	Percentage of variation	Fixation indices
Among regions	1	52.221	−0.25575Va	−0.83	*F*_CT_ = −0.00834^ns^ (*P* ≤ 0.0001)
Among populations within regions	14	1418.562	3.06055Vb	9.98	*F*_SC_ = 0.09143 (*P* ≤ 0.0001)
Within populations	369	10257.909	27.87262Vc	90.86	*F*_ST_ = 0.09894 (*P* ≤ 0.0001)

Total	384	11727.909	30.67742		

**Table 5:** Indices of genetic differentiation *F*_*SC*_ showed by the groups of isolates when comparing each one with the others according to varieties and geographic origins.

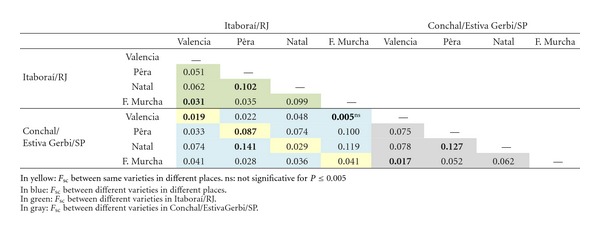

**Table 6:** Indices of genetic differentiation *F*_*ST*_ showed by isolates groups when compared each one with the others. The number of haplotypes shared between populations of *G. citricarpa *of this work are in parenthesis.

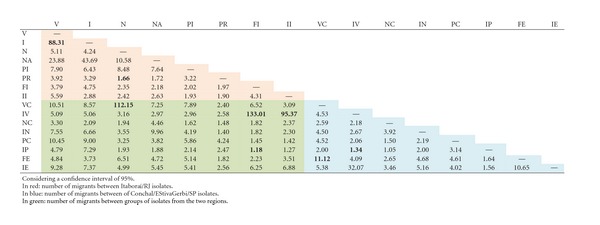

**Table 7:** Number of migrant haplotypes per generation between populations of *G. citricarpa* from different samplings, varieties and geographic origins.