# Supplementary material for: Genetic Diversity and Population Differentiation of the Causal Agent of Citrus Black Spot in Brazil
Source: ScientificWorldJournal. 2012 May 15;2012:368286. doi: 10.1100/2012/368286 (PMC3362019; doi:10.1100/2012/368286)
Supplement: Supplementary file 1 — This Table contains the Guignardia mangiferae isolates used for this study. ITS1-5.8S-ITS2 DNA region of each isolate was amplified in order to perform the population genetic structure described on this research. The DNA sequence was deposited in GenBank and received an Acession Number presented on Table. [file 368286.f1.docx]

Supplementar data: *G. citricarpa* isolates from Itaboraí/Rio de Janeiro State, Brazil.

| Isolate//GenBank ID | Isolate//GenBank ID | Isolate//GenBank ID | Isolate//GenBank ID | Isolate//GenBank ID | Isolate//GenBank ID | Isolate//GenBank ID | Isolate//GenBank ID |
| --- | --- | --- | --- | --- | --- | --- | --- |
| V-1//JQ397200 | I-1//JQ397105 | N-1//JQ397033 | NA-1//JQ397057 | PI-1//JQ397201 | PR-1//JQ397249 | FI-1//JQ396937 | II-1//JQ396985 |
| V-2//JQ397199 | I-2//JQ397106 | N-2//JQ397034 | NA-2//JQ397058 | PI-2//JQ397202 | PR-12//JQ397250 | FI-2//JQ396938 | II-2//JQ396986 |
| V-3//JQ397198 | I-3//JQ397107 | N-3//JQ397035 | NA-3//JQ397059 | PI-3//JQ397203 | PR-11//JQ397251 | FI-3//JQ396939 | II-3//JQ396987 |
| V-4//JQ397197 | I-4//JQ397108 | N-4//JQ397036 | NA-4//JQ397060 | PI-4//JQ397204 | PR-10//JQ397252 | FI-4//JQ396940 | II-4//JQ396988 |
| V-5//JQ397196 | I-5//JQ397109 | N-5//JQ397037 | NA-6//JQ397061 | PI-5//JQ397205 | PR-9//JQ397253 | FI-5//JQ396941 | II-5//JQ396989 |
| V-6//JQ397195 | I-6//JQ397110 | N-6//JQ397038 | NA-7//JQ397062 | PI-6//JQ397206 | PR-8//JQ397254 | FI-6//JQ396942 | II-6//JQ396990 |
| V-7//JQ397194 | I-7//JQ397111 | N-7//JQ397039 | NA-8//JQ397063 | PI-7//JQ397207 | PR-6//JQ397255 | FI-7//JQ396943 | II-7//JQ396991 |
| V-8//JQ397193 | I-8//JQ397112 | N-8//JQ397040 | NA-9//JQ397064 | PI-8//JQ397208 | PR-5//JQ397256 | FI-8//JQ396944 | II-8//JQ396992 |
| V-9//JQ397177 | I-9//JQ397113 | N-9//JQ397041 | NA-10//JQ397065 | PI-9//JQ397209 | PR-4//JQ397257 | FI-9//JQ396945 | II-13//JQ396993 |
| V-10//JQ397178 | I-10//JQ397114 | N-10//JQ397042 | NA-11//JQ397066 | PI-10//JQ397210 | PR-3//JQ397258 | FI-10//JQ396946 | II-14//JQ396994 |
| V-11//JQ397179 | I-11//JQ397115 | N-14//JQ397043 | NA-13//JQ397067 | PI-11//JQ397211 | PR-2//JQ397259 | FI-11//JQ396947 | II-15//JQ396995 |
| V-12//JQ397180 | I-12//JQ397116 | N-15//JQ397044 | NA-14//JQ397068 | PI-12//JQ397212 | PR-7//JQ397260 | FI-12//JQ396948 | II-16//JQ396996 |
| V-13//JQ397181 | I-13//JQ397117 | N-16//JQ397045 | NA-15//JQ397069 | PI-13//JQ397213 | PR-13//JQ397261 | FI-13//JQ396949 | II-18//JQ396997 |
| V-14//JQ397182 | I-14//JQ397118 | N-17//JQ397046 | NA-16//JQ397070 | PI-14//JQ397214 | PR-14//JQ397262 | FI-14//JQ396950 | II-19//JQ396998 |
| V-15//JQ397183 | I-15//JQ397119 | N-18//JQ397047 | NA-18//JQ397071 | PI-15//JQ397215 | PR-15//JQ397263 | FI-15//JQ396951 | II-20//JQ396999 |
| V-16//JQ397184 | I-16//JQ397120 | N-19//JQ397048 | NA-19//JQ397072 | PI-16//JQ397216 | PR-18//JQ397264 | FI-16//JQ396952 | II-22//JQ397000 |
| V-17//JQ397185 | I-17//JQ397121 | N-20//JQ397049 | NA-20//JQ397073 | PI-17//JQ397217 | PR-20//JQ397265 | FI-17//JQ396953 | II-23//JQ397001 |
| V-18//JQ397186 | I-18//JQ397122 | N-22//JQ397050 | NA-22//JQ397074 | PI-18//JQ397218 | PR-21//JQ397266 | FI-18//JQ396954 | II-25//JQ397002 |
| V-19//JQ397187 | I-19//JQ397123 | N-23//JQ397051 | NA-23//JQ397075 | PI-20//JQ397219 | PR-23//JQ397267 | FI-19//JQ396955 | II-26//JQ397003 |
| V-20//JQ397188 | I-20//JQ397124 | N-24//JQ397052 | NA-24//JQ397076 | PI-21//JQ397220 | PR-24//JQ397268 | FI-20//JQ396956 | II-27//JQ397004 |
| V-21//JQ397189 | I-21//JQ397125 | N-25//JQ397053 | NA-25//JQ397077 | PI-22//JQ397221 | PR-25//JQ397269 | FI-21//JQ396957 | II-29//JQ397005 |
| V-22//JQ397190 | I-22//JQ397126 | N-26//JQ397054 | NA-26//JQ397078 | PI-24//JQ397222 | PR-27//JQ397270 | FI-22//JQ396958 | II-31//JQ397006 |
| V-23//JQ397191 | I-23//JQ397127 | N-27//JQ397055 | NA-27//JQ397079 | PI-26//JQ397223 | PR-28//JQ397271 | FI-23//JQ396959 | II-33//JQ397007 |
| V-24//JQ397192 | I-24//JQ397128 | N-28//JQ397056 | NA-28//JQ397080 | PI-28//JQ397224 | PR-31//JQ397272 | FI-24//JQ396960 | II-34//JQ397008 |

Supplementar data – isolates from Estiva Gerbi/Conchal/São Paulo State, Brazil.

| Isolate//GenBank ID | Isolate//GenBank ID | Isolate//GenBank ID | Isolate//GenBank ID | Isolate//GenBank ID | Isolate//GenBank ID | Isolate//GenBank ID | Isolate//GenBank ID |
| --- | --- | --- | --- | --- | --- | --- | --- |
| VC-1//JQ397153 | IV-1//JQ397129 | NC-1//JQ397009 | IN-1//JQ397081 | PC-1 // FJ769669 | IP-1//JQ397225 | FE-1//JQ396913 | IE-1//JQ396961 |
| VC-2//JQ397154 | IV-2//JQ397130 | NC-2//JQ397010 | IN-2//JQ397082 | PC-2 // FJ769675 | IP-2//JQ397226 | FE-2//JQ396914 | IE-2//JQ396962 |
| VC-3//JQ397155 | IV-3//JQ397131 | NC-3//JQ397011 | IN-3//JQ397083 | PC-3 // FJ769676 | IP-3//JQ397227 | FE-3//JQ396915 | IE-3//JQ396963 |
| VC-4//JQ397156 | IV-4//JQ397132 | NC-4//JQ397012 | IN-4//JQ397084 | PC-4 // FJ769679 | IP-4//JQ397228 | FE-4//JQ396916 | IE-5//JQ396964 |
| VC-5//JQ397157 | IV-5//JQ397133 | NC-5//JQ397013 | IN-5//JQ397085 | PC-5 // FJ769680 | IP-5//JQ397229 | FE-5//JQ396917 | IE-6//JQ396965 |
| VC-6//JQ397158 | IV-6//JQ397134 | NC-6//JQ397014 | IN-6//JQ397086 | PC-6 // FJ769677 | IP-6//JQ397230 | FE-6//JQ396918 | IE-7//JQ396966 |
| VC-7//JQ397159 | IV-7//JQ397135 | NC-7//JQ397015 | IN-7//JQ397087 | PC-7 // FJ769682 | IP-7//JQ397231 | FE-7//JQ396919 | IE-8//JQ396967 |
| VC-8//JQ397160 | IV-8//JQ397136 | NC-8//JQ397016 | IN-8//JQ397088 | PC-9 // FJ769678 | IP-8//JQ397232 | FE-8//JQ396920 | IE-9//JQ396968 |
| VC-9//JQ397161 | IV-9//JQ397137 | NC-9//JQ397017 | IN-9//JQ397089 | PC-10 // FJ769659 | IP-9//JQ397233 | FE-10//JQ396921 | IE-11//JQ396969 |
| VC-10//JQ397162 | IV-10//JQ397138 | NC-10//JQ397018 | IN-10//JQ397090 | PC-11 // FJ769660 | IP-11//JQ397234 | FE-11//JQ396922 | IE-12//JQ396970 |
| VC-11//JQ397163 | IV-11//JQ397139 | NC-11//JQ397019 | IN-11//JQ397091 | PC-12 // FJ769661 | IP-12//JQ397235 | FE-12//JQ396923 | IE-13//JQ396971 |
| VC-12//JQ397164 | IV-12//JQ397140 | NC-13//JQ397020 | IN-12//JQ397092 | PC-13 // FJ769662 | IP-13//JQ397236 | FE-13//JQ396924 | IE-14//JQ396972 |
| VC-13//JQ397165 | IV-13//JQ397141 | NC-14//JQ397021 | IN-13//JQ397093 | PC-14 // FJ769663 | IP-14//JQ397237 | FE-14//JQ396925 | IE-15//JQ396973 |
| VC-14//JQ397166 | IV-14//JQ397142 | NC-15//JQ397022 | IN-14//JQ397094 | PC-15 // FJ769664 | IP-16//JQ397238 | FE-15//JQ396926 | IE-16//JQ396974 |
| VC-15//JQ397167 | IV-15//JQ397143 | NC-16//JQ397023 | IN-15//JQ397095 | PC-16 // FJ769665 | IP-19//JQ397239 | FE-16//JQ396927 | IE-18//JQ396975 |
| VC-16//JQ397168 | IV-16//JQ397144 | NC-18//JQ397024 | IN-16//JQ397096 | PC-17 // FJ769666 | IP-23//JQ397240 | FE-17//JQ396928 | IE-19//JQ396976 |
| VC-17//JQ397169 | IV-17//JQ397145 | NC-19//JQ397025 | IN-17//JQ397097 | PC-18 // FJ769667 | IP-24//JQ397241 | FE-18//JQ396929 | IE-21//JQ396977 |
| VC-18//JQ397170 | IV-18//JQ397146 | NC-20//JQ397026 | IN-18//JQ397098 | PC-19 // FJ769668 | IP-25//JQ397242 | FE-19//JQ396930 | IE-22//JQ396978 |
| VC-19//JQ397171 | IV-19//JQ397147 | NC-21//JQ397027 | IN-19//JQ397099 | PC-20 // FJ769670 | IP-26//JQ397243 | FE-20//JQ396931 | IE-24//JQ396979 |
| VC-20//JQ397172 | IV-20//JQ397148 | NC-22//JQ397028 | IN-20//JQ397100 | PC-21 // FJ769671 | IP-28//JQ397244 | FE-21//JQ396932 | IE-25//JQ396980 |
| VC-21//JQ397173 | IV-21//JQ397149 | NC-23//JQ397029 | IN-22//JQ397101 | PC-22 // FJ769681 | IP-29//JQ397245 | FE-22//JQ396933 | IE-26//JQ396981 |
| VC-22//JQ397174 | IV-22//JQ397150 | NC-25//JQ397030 | IN-23//JQ397102 | PC-24 // FJ769672 | IP-30//JQ397246 | FE-23//JQ396934 | IE-27//JQ396982 |
| VC-23//JQ397175 | IV-23//JQ397151 | NC-26//JQ397031 | IN-24//JQ397103 | PC-26 // FJ769673 | IP-31//JQ397247 | FE-24//JQ396935 | IE-31//JQ396983 |
| VC-24//JQ397176 | IV-24//JQ397152 | NC-28//JQ397032 | IN-25//JQ397104 | PC-27 // FJ769674 | IP-32//JQ397248 | FE-25//JQ396936 | IE-32//JQ396984 |
